# Scanning Transmission Soft X‐Ray Microscopy Probes Topical Drug Delivery of Rapamycin Facilitated by Microneedles

**DOI:** 10.1002/cphc.202400819

**Published:** 2024-11-19

**Authors:** J. A. Laux, T. Ohigashi, M. R. Bittermann, T. Araki, H. Yuzawa, F. Rancan, A. Vogt, E. Rühl

**Affiliations:** ^1^ Physikalische Chemie Freie Universität Berlin, Arnimallee 22 14195 Berlin Germany; ^2^ UVSOR Synchrotron Facility Institute for Molecular Science, Myodaiji Okazaki 444-8585 Japan; ^3^ Charité - Universitätsmedizin 10117 Berlin Germany

**Keywords:** X-ray microscopy, human skin, drug delivery, microneedles

## Abstract

Scanning Transmission X‐ray microscopy (STXM) is a sensitive and selective probe for the penetration of rapamycin which is topically applied to human skin *ex vivo* and is facilitated by skin treatment with microneedles puncturing the skin. Inner‐shell excitation serves as a selective probe for detecting rapamycin by changes in optical density as well as linear combination modeling using reference spectra of the most abundant species. The results indicate that mechanical damage induced by microneedles allows this drug to accumulate in the stratum corneum without reaching the viable skin layers. This is unlike intact skin which shows no drug penetration at all and underscores the mechanical impact of microneedle skin treatment. These results are compared to drug penetration profiles of other drugs highlighting the importance of skin barriers. High spatial resolution studies also indicate that the lipophilic drug rapamycin is observed in corneocytes. Attempts in data evaluation are reported to probe rapamycin also in the lipid layers between the corneocytes, which was not accomplished before. These results are compared to recent results on rapamycin uptake in skin where barrier impairment was induced by pre‐treatment with the enzyme trypsin and drug formulations leading to occlusion.

## Graphical abstract

Human skin treated with microneedles facilitates penetration of topically applied drugs that cannot penetrate intact skin, such as rapamycin. This drug is selectively probed by scanning transmission X‐ray microscopy and specific data evaluation approaches.

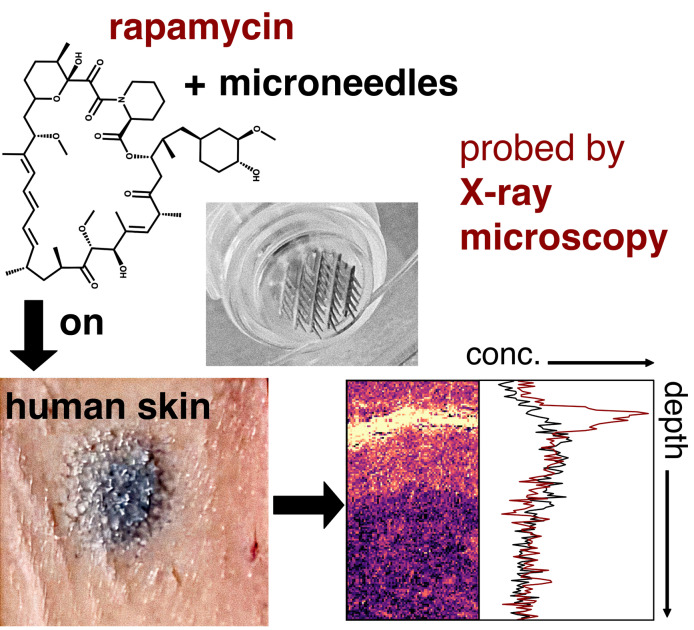

## Introduction

Scanning transmission X‐ray microscopy (STXM) and related approaches employ tunable soft X‐rays for spatially resolved spectromicroscopy studies. It is an established approach for label‐free probing of various samples.[^1^, ^2^, ^3^, ^4^, ^5^, ^6^, ^7^] This includes samples of importance to materials science, biological matter, as well as drug delivery systems. Its strength lies in the use of tunable soft X‐rays delivered from synchrotron radiation sources allowing for element‐selective excitations and exploiting chemical shifts of resonant transitions, for probing the chemical environment of the absorber with spatial resolution on the nanoscale. Recently, various approaches in spectromicroscopy relevant to drug delivery have been reviewed.^[8]^ Among these are experimental approaches relying on sensitive labels, such as fluorescence, as well as label‐free approaches that utilize the intrinsic spectroscopic properties of the species to be probed with high spatial resolution. Of crucial importance to STXM is data reduction for deriving from the spatially resolved spectroscopic data details on the spatial distribution of the species of interest. A variety of approaches exist, which have been reviewed recently.^[9]^ Most important is the use of specific software for treating the raw data, e. g., aXis2000,^[10]^ so that changes in optical density and drifts in image stacks can be evaluated. We have recently developed an approach that is based on linear combination modeling of reference spectra[^11^, ^12^] and is based on singular value decomposition.^[13]^ Furthermore other approaches, such as cluster analyses and principal component analyses have been used, e. g., by employing the program MANTiS.^[14]^

Topical drug delivery is of central importance to dermal applications, as skin is the largest human organ that can be affected by a variety of diseases, most important are inflammatory skin diseases, such as psoriasis and atopic dermatitis.[^15^, ^16^] Investigations of topical drug delivery applied to skin have the advantage that the transport path of the drug is short until it reaches its site of action in the viable parts of the skin, which is of the order of 150 μm. This is fully compatible with the size of samples that can be investigated by STXM, provided the sample thickness is sufficiently thin allowing for transmission of soft X‐rays. Previous work has shown that soft X‐rays can penetrate skin samples with a thickness of the order of 300–500 nm,^[6]^ which is demanding for sample preparation, as this is significantly thinner than biological samples prepared for optical microscopy^[17]^ and thicker than electron microscopy samples.^[18]^ Another limitation in sample preparation is the occasional access to soft X‐ray microscopes located at highly demanded synchrotron radiation facilities. This is prohibitive for studies on fresh biological samples and requires sample preparation to make them last until the access to the X‐ray microscope is granted. Specifically, biological samples are embedded in polymers, such as EPON, a technique that is also used in electron microscopy.^[19]^

Microneedles made of metals or polymers have been used for topical intra‐ and transdermal drug delivery or vaccination.[^20^, ^21^, ^22^, ^23^, ^24^, ^25^, ^26^, ^27^, ^28^] They consist of arrays of micron sized needles piercing the upper skin layers and can be formed from different materials, shape, and length with or without a bore. Their shape and choice of materials are of crucial importance and they might even dissolve.[^29^, ^30^] The use of microneedles is manifold, it is mainly pain‐free in use specifically for skin treatment, and has developed into commercial products.^[31]^ Clinical use of microneedles has been reviewed before to be useful for numerous applications reaching from diagnostic to therapeutic applications as well as use in cosmetics.^[32]^

Rapamycin (sirolimus) is a highly potent drug that is an mTOR inhibitor (mammalian target of rapamycin). Various medical applications of the immunosuppressive drug are known reaching from cancer treatment,^[33]^ to use in organ transplantations.^[34]^ There are also applications in dermatology, as recently reviewed with systemic and topical applications.^[35]^

The motivation for this study is that it is known that rapamycin cannot penetrate intact skin, as was also evidenced by STXM.^[36]^ This finding was rationalized in terms of the 500 Da rule,^[37]^ as the molecular weight M of rapamycin is 914.187 Da. Here, one important barrier preventing drug penetration is the stratum corneum, the top horny layer of skin, and the tight junctions located in the stratum granulosum.^[38]^ Evidently, tight junctions act as an inside‐out as well as an outside‐in barrier. For topical drug delivery properties of the outside‐in barriers are of importance and have been studied by tracers.^[39]^ As a result, tight junctions play an important role for suppression of molecular penetration. Moreover, properties of lower skin barriers, such as the basal membrane, have been investigated before using dexamethasone as a model drug.[^40^, ^41^] These studies reveal that for hydrophobic drugs, which include dexamethasone as well as rapamycin, the basal membrane is a high free energy barrier, which is unlike the stratum corneum that is a diffusion barrier. This implies that hydrophobic drugs are best accommodated in the more lipophilic viable epidermis than the dermis. In previous studies, the barrier of the stratum corneum and the stratum granulosum (tight junctions) has been modified enzymatically and by using occluding formulations.[^11^, ^36^] Enzymatically‐induced partial barrier disruptions were induced by low concentrations of trypsin, a serine protease. It was shown that after a short treatment time of 10 and 100 min prior to topical drug administration, rapamycin can only penetrate the stratum corneum. After 1000 min of topical trypsin treatment, one observes even in the viable epidermis the penetration of topically applied rapamycin, implying that the tight junctions become leaky due to the initial trypsin treatment.^[36]^ If petrolatum‐based formulations of rapamycin are topically applied, which are known to induce occlusion, this also leads to the penetration of the stratum corneum compared to untreated skin, whereas no drug is observed in the viable layers.^[11]^ This implies that occlusion does not damage the tight junctions. This knowledge motivated the present study, where instead of enzyme‐ or occlusion‐induced changes in skin barrier, the mechanical damage of the top skin barrier is investigated by applying microneedle treatment immediately after drug application using a commercial device. The length of the microneedles was adjusted to 250 μm to reach upper and mid‐viable parts of the skin and damage the tight junctions. This is unlike earlier efforts which used considerably longer needles up to 900 μm that may cause more profound changes and alterations of the transport of substances into the skin.^[42]^

## Experimental

Tunable synchrotron radiation in the O 1s‐regime was received from the beamline BL4 U at the UVSOR‐III Synchrotron (Institute for Molecular Science, Okazaki, Japan). UVSOR‐III was operated at 750 MeV and 300 mA in the top‐up injection mode. The operation parameters of the STXM (Research Instruments, ex Bruker) were identical to those reported before.^[36]^ The photon energy scale in the O 1s excitation regime (520 – 565 eV) was established by using the known transition energies of the O 1 s→π*‐transitions, such as CO_2_ and rapamycin.^[6]^ The energy resolution was ∼60 meV and the spot size of the X‐ray photons on the sample was 74±3 nm. The STXM data were collected in the ‘on‐the‐fly mode’ at pixel sizes of 1 μm for overview images of the entire samples and reached down to 200–250 nm and 50 nm, respectively, for more detailed scans. The typical dwell time for each pixel was 2 ms. Scan sizes were 150 μm x 400 μm (overview scans), 50 μm x 10 μm, and ~16 μm x 3 μm, respectively. Each stack of data contained 134 maps taken at photon energies between 520 eV and 565 eV, except for overview scans that covered only 11–13 energy points between 530.3 eV and 531.5 eV, as these large micrographs were too time consuming for the limited beam time. Near‐edge features at the O 1s‐edge were taken at small energy steps of 0.1 eV. The pre‐edge‐ and post‐edge‐continua were taken with larger step widths of 0.5 eV and 0.607 eV, respectively, as there are no narrow absorption features observed.

Details on data reduction are found in the Supporting Information (Section 1). Briefly, changes in optical density due to absorption of the species of interest were obtained according to the Beer‐Lambert law, similarly to earlier work^[6]^: *ln(I_0_/I)=σ ⋅ c ⋅ d*, where σ is the absorption cross section, *c* is the concentration of the absorber, and *d* is the thickness of the sample, which can be determined, e. g., by atomic force microscopy.^[6]^ Absolute absorption cross sections were derived at 560 eV using tabulated reference data.^[43]^ Alternatively, linear combination modeling was used in combination with reference spectra, similar to previous work.[^11^, ^36^] Briefly, reference spectra were extracted for the dominant species, which includes rapamycin, EPON, an averaged spectrum of human skin, as well as components corresponding to the O 1 s→π*‐transition and the O 1 s→σ*‐transition (see Supporting Information, Figure S1).

Human skin samples were prepared as follows: *ex vivo* skin samples came from the upper arm of a female donor after informed consent. This research was conducted in accordance with the Declaration of Helsinki guidelines and was approved by the Ethics Committee of Charité ‐ Universitätsmedizin Berlin (Germany) (approval EA1/135/06, renewed in January 2019). Drug formulations were made of hydroxy ethyl cellulose (HEC, 2.5 % w/v) gel that was prepared from a solution of rapamycin (LC Laboratories, Woburn, MA, U.S.A.) dissolved in 70 % ethanol, yielding a drug concentration of 1 mg/mL.

The skin samples were cut into 1.5×1.5 cm pieces and were pinned on Styrofoam plates to avoid contraction during drug treatment. Then, 40 μL of the drug formulation were placed topically on the skin samples. Previous work indicated that such formulations do not penetrate intact skin, even in the top layers of the stratum corneum no uptake was detected.^[36]^ Subsequently, the skin samples were treated with a commercial dermatological microneedle pen (derma pen) (Auto‐Stamp Motorized Meso Machine)^[44]^ containing 36 needles of circular shape adjusted to reach a microneedle penetration depth up to 250 μm, as shown in Figure S2. The tip size is 15±5 μm (see Figure S2(b)). The device was operated for 10 s with 18000 RPM at a given position, corresponding to 300 hits ⋅ s^−1^. The distance between the needles in one row is about 0.5 mm, which appears to be too far apart to have a sufficient number of damaged regions for STXM studies, considering that a skin section has a thickness of ca. 300 nm and a width of ca. 0.5 mm. Therefore, the derma pen was rotated by approximately 45° every 5 s and 10 series of 4 rotations were applied. Initial studies using the dye trypan blue for visualization of the mechanical impact indicated that a uniform staining of the skin samples was achieved in this way while no severe mechanical damage of the skin was observed (see Supporting Information Figure S2(d), (e)). This protocol was adopted for drug‐treated skin and subsequently the skin samples were left for 10, 100, and 1000 min to allow the drug to penetrate the mechanically damaged skin. Subsequently, the remaining formulation was removed, and the skin did not show visible damage due to microneedle impact. The skin was cut into 1x1 mm pieces and was fixed in 2.5 % glutaraldehyde and 1 % cacodylate buffer. The next steps were dehydration and embedding in EPON resin, as described in previous work.[^6^, ^11^, ^45^, ^46^, ^47^] The samples were cut using an ultramicrotome into ca. 300 nm thick slices having an area of ca. 500×500 μm. They were prepared as duplicates, corresponding to neighboring slices. All skin samples were placed on silicon nitride (Si_3_N_4_) windows with a thickness of 100 nm (Silson) and were finally characterized by optical microscopy (Nikon, MM‐400/LU) with magnifications up to ×100 (see Supporting Information Figure S3). These micrographs served to select regions to be investigated by STXM, where preferably the stratified skin layers are oriented parallel to the silicon frame of the silicon nitride windows and no damage of the skin samples should be visible. Figure S3 shows the higher spatial resolution of STXM, which is also confirmed by supplementary atomic force microscopy (AFM) experiments. The samples that were exposed for 10 and 100 min to the drug formulation and the untreated reference samples fulfilled these requirements, whereas both samples exposed for 1000 min to the drug formulation showed a crack separating the stratum corneum from the viable epidermis, which leads to artefacts in data evaluation. For STXM studies, the skin surface was vertically oriented as the STXM scan direction was horizontal. Then, each scanned line corresponds to a depth profile.

## Results and Discussion

**Figure 1:**
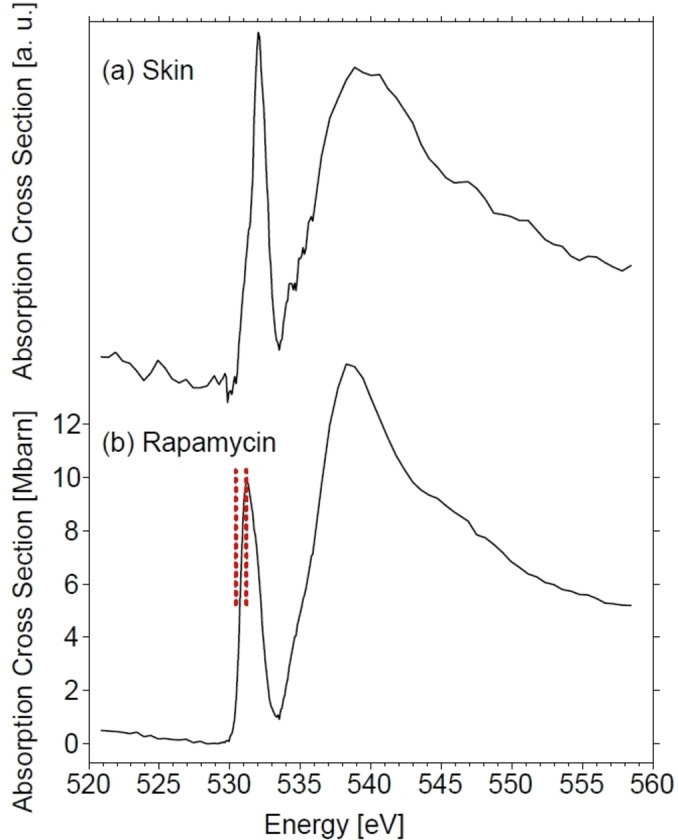
Comparison of the O 1s spectrum of (a) fixed human skin and (b) rapamycin. The vertical dashed red lines indicate the photon energies that were used to determine the optical density for probing rapamycin by changes in optical density. See text for further details.

**Figure 2:**
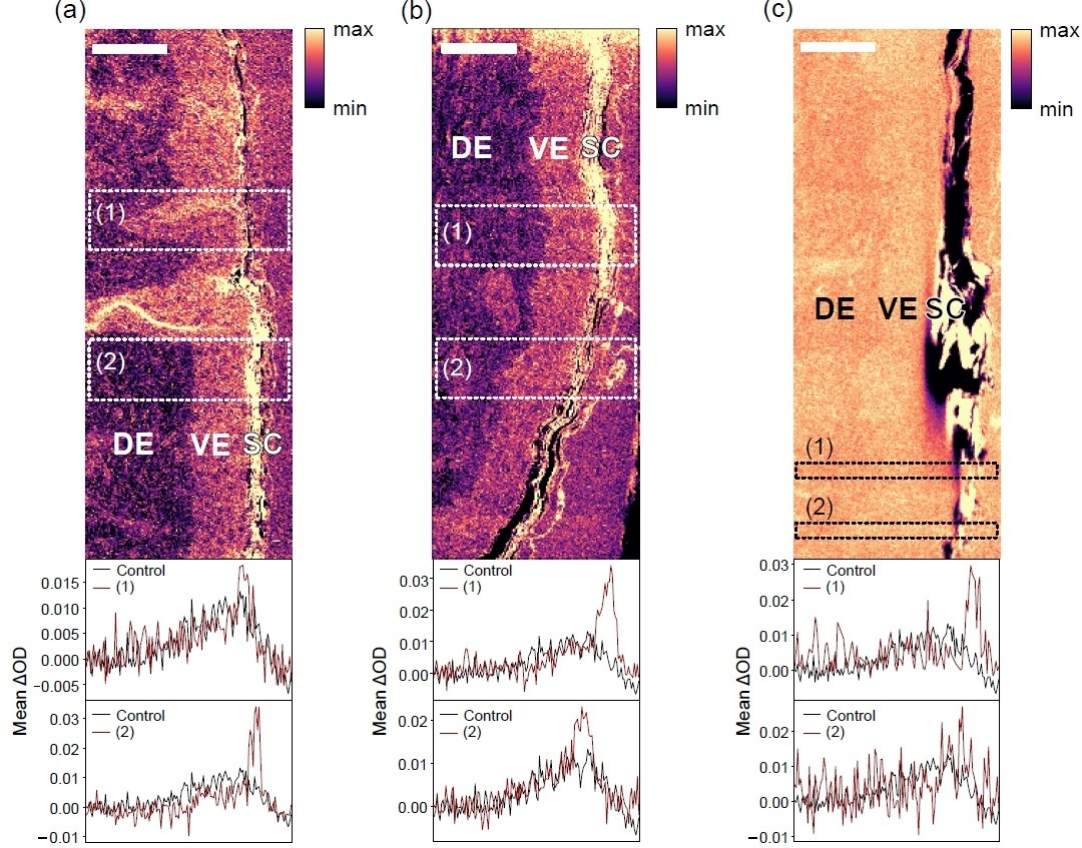
Overview micrographs of microneedle‐treated skin samples that were exposed to a topically applied rapamycin formulation for (a) 10 min, (b) 100 min, and (c) 1000 min (photon energy: 531.4 eV, range: 5–95 % clipped data). The dashed regions marked by numbers the evaluated regions with respect to changes in optical density between 531.2 eV (maximum of O 1 s→π*‐transition of rapamycin) and 530.5 eV for probing rapamycin (cf. Figure 1). All curves were compared to an untreated control (black curves in the depth profiles, cf. Figure S4). The scale bars correspond to 50 μm. DE: Dermis, VE: Viable epidermis, SC: Stratum corneum. See text for further details.

**Figure 3:**
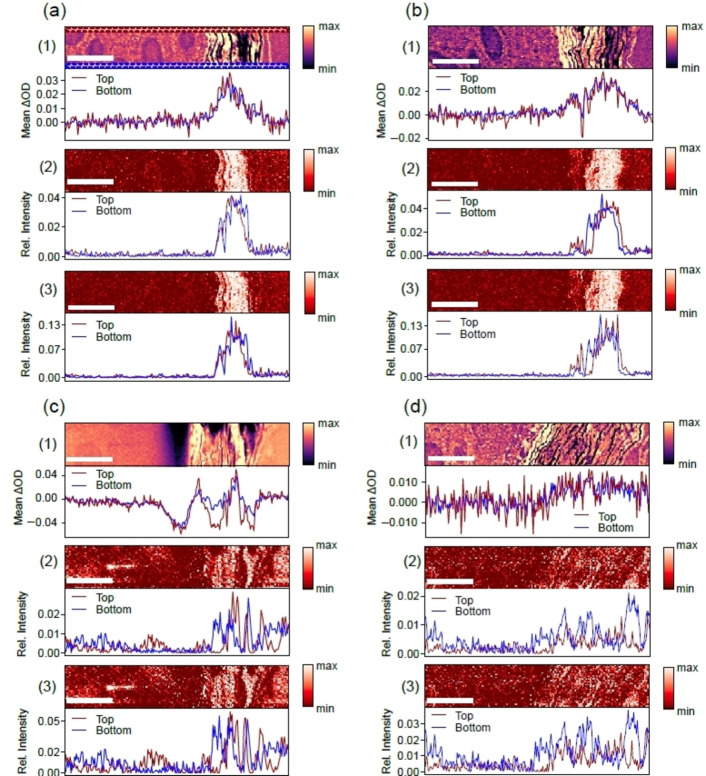
(a) 10 min treated skin sample; (b) 100 min treated skin sample; (c) 1000 min treated skin sample as well as (d) results from an untreated reference skin. Each Figure consists of: (1) X‐ray micrograph taken at 532.0 eV (range: 5–95 % clipped data) (top) and changes in optical density between 531.2 eV and 530.2 eV (bottom); (2) linear combination modeling (LCM)‐component map for rapamycin (top), where only the reference spectra were normalized to the O 1 s→σ*‐resonance; depth profile of the LCM map (bottom); (3) LCM‐component maps for rapamycin, where the reference spectra and each pixel of the micrographs were normalized to the O 1 s→σ*‐resonance (top); depth profile of the LCM map (bottom). The depth profiles were derived for ‘top’ and ‘bottom’ from the indicated colored rectangles indicated in (a) region (1). The scale bars correspond to 10 μm.

**Figure 4:**
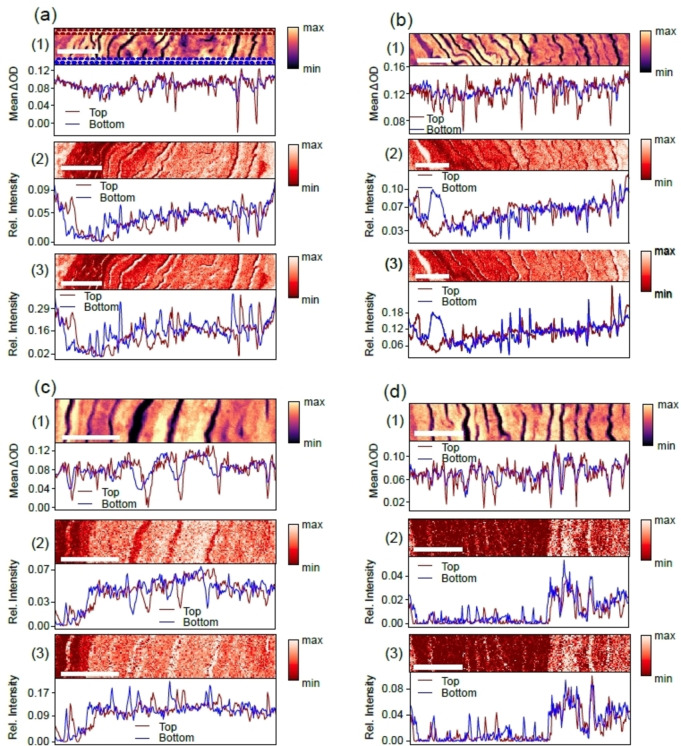
Spatial distribution of rapamycin in the stratum corneum, as derived from linear combination modeling at high spatial resolution: (a) 10 min treated skin sample; (b) 100 min treated skin sample; (c) 1000 min treated skin sample; (d) untreated reference skin. For the analysis, the upper and lower 10 pixels of the width of the scans were considered, as illustrated by dashed lines in (a). (1) Top: X‐ray micrograph taken at 532.0 eV (range: 5–95 % clipped data). Bottom: Changes in optical density between 531.2 eV and 530.2 eV; (2) Top: linear combination modeling (LCM)‐component map for rapamycin, where only the reference spectra were normalized to the O 1 s→σ*‐resonance. Bottom: Depth profile of the linear combination modeling map; (3) Top: linear combination modeling‐component map for rapamycin, where the reference spectra and each pixel of the micrographs were normalized to the O 1 s→σ*‐resonance. Bottom: Depth profile of this map. The scale bars correspond to 3 μm.

Figure 1 shows the reference spectra of (a) fixed human skin and (b) rapamycin that are used for data evaluation. Note that the quality of the O 1s‐absorption of rapamycin is significantly improved compared to previous work^[36]^ and shows more clearly that the O 1 s→π*‐transition of rapamycin peaking at 531.2 eV is slightly redshifted relative to that of fixed human skin peaking at 532.0 eV. This allows us to determine from evaluation of the optical density the relative local concentration of the drug. Also note that the absorption energies used for this evaluation were slightly modified compared to previous work,^[36]^ corresponding to 531.2 eV and 530.5 eV for the overview scans (cf. Figure 2), respectively, indicated by vertical red lines in Figure 1. Note that slightly different values were used for determining changes in optical density in Figures 3 and 4 (531.2 eV and 530.2 eV). It is impossible to fully avoid by this approach cross sensitivities from untreated skin, especially for low drug concentrations (cf. Supporting Information Figure S4). However, the modified energies yield an enhanced optical density for rapamycin than using the previously chosen ones (531.1 eV and 530.74 eV).^[36]^ The other broad spectral feature peaking at 538.5 eV is due to the O 1 s→σ*‐transition. This transition energy is by 1 eV lower in energy than previously reported, as the present spectral quality reported in this work is improved compared to earlier results.^[36]^ The absolute cross section peaks in this energy regime at the O 1 s→σ*‐transition with a value of 14.5±1 Mbarn, which is derived from the atomic absorption cross section^[43]^ and is comparable in magnitude to the previously reported value.^[36]^ The O 1s ‐ spectrum of fixed human skin is similar in shape as reported before.^[6]^ However, the O 1 s→π*‐transition is slightly more intense than in previous work,[^6^, ^45^] which is likely due to a different origin of the skin samples and small changes in the fixation protocol. This might explain variations in unsaturated to saturated moieties, corresponding to the intensity ratio of the O 1 s→π*‐transition compared to the O 1 s→σ*‐transition. Note that the absolute cross section of fixed human skin cannot be determined, as the elemental composition is not exactly known, which is unlike rapamycin with its molecular composition C_51_H_79_NO_13_, M =914.17 Da.

Figure 2 shows a series of skin samples initially treated with microneedles, as described in the Experimental Section, and then left at 37 °C for (a) 10, (b) 100, and (c) 1000 min so that the drug can penetrate the samples for the indicated time periods. The size of the scanned areas was chosen to be wide, as it was initially unclear if the investigated samples show any evidence of drug penetration, as the penetration depth of the needles was not known beforehand due to the elasticity of the skin and the mechanical barrier of the stratum corneum. We note that no punctured regions were visible in the microneedle treated skin, which is expected due the large distance between the needles and the small number of samples that was produced and investigated due to limited beam time.

It is known that no rapamycin can penetrate intact skin samples.^[36]^ Therefore, detecting any rapamycin in the skin samples would be an indication for the impact of microneedle treatment. The micrographs shown in Figure 2(a)–(c) cover an area of 150 μm depth and 400 μm width, at a thickness of ca. 300 nm. The X‐ray micrographs were taken at 531.4 eV and show the highest contrast in this measurement represented on a false color scale. To increase clarity for structural differences, only the 5–95 % clipped data have been used for visualization. Such presentation clearly shows the vertical morphology of the skin samples near the surface. The skin is oriented in a way that the skin surface is found on the right‐hand side of each X‐ray micrograph. Above the skin surface (right‐hand side of Figures 2(a)‐(c)) one only finds EPON that is used for embedding the skin samples (see Experimental Section). The stratum corneum is found as a thin yellow structure of 10–20 μm thickness followed with increasing depth by the viable epidermis (VE) and the dermis (DE). These are clearly separated by color contrast, due to different morphology and chemical composition of the skin regions. Also, there are some defects visible. Figure 2(a) (10 min exposure time) indicates funnel‐like structures that are identified as hair follicles and vary the depth of the viable epidermis in these regions. Figure 2(b) (100 min exposure time) does not show such structures, here the border between the viable epidermis and the dermis is much less structured so that the viable epidermis has an almost constant thickness. There is evidence for cracks at the stratum corneum in the lower part of the micrograph (cf. Figure 2(b)), which is indicated by black color. Figure 2(c) shows a crack through the entire skin sample at the stratum corneum, which is likely due to the long exposure time of 1000 min that weakens the mechanical stability of the skin and leads to damage upon fixation.

The samples shown in Figure 2(a)–(c) were investigated in the region of the O 1 s→π*‐transition at 11 and 13 photon energies between 530.3 and 531.5 eV, respectively, for probing the spatial distribution of rapamycin by changes in optical density. This stack of images is used to determine changes in optical density at two selected photon energies (531.2 eV and 530.5 eV, respectively) where rapamycin is efficiently probed (cf. Figure 1).^[36]^ They were chosen for determining rapamycin depth profiles over the entire sample, since it was initially not clear, where the drug can be found after microneedle impact. Here, we show, as typical examples, only two sub‐sections per experiment, as indicated in Figure 2 by dashed rectangles, for determining if the microneedle approach may inject the drug deep into viable skin regions. These sub‐sections were mostly selected for regions where no significant morphological changes within the stratified structure of the skin sections occur, i. e., where the skin layers are sufficiently parallel to each other. In Figure 2(a) region 1 corresponds to a feature that is due to a hair follicle, whereas region 2 shows horizonal layers parallel to the skin surface. This proves to yield the clearest results for the analysis outlined below, as inclined or bended skin layers may distort the depth profiles of rapamycin. Note that in Figure 2(c), corresponding to 1000 min exposure time, regions of the smallest defects were selected. Previous work also indicated that there are cross sensitivities from the fixed skin matrix,^[36]^ so that an unstructured background signal is expected to occur. Note that previous work on different skin samples and topically applied drugs probed by STXM in comparison with other spectromicroscopy and analytical techniques indicates that reliable results are derived despite cross sensitivities.[^48^, ^49^] The background signal is determined from an untreated reference sample that is included as a black curve in the lower part of Figure 2(a)–(c). The evaluated data from the reference sample are plotted in Figure S4. The red curves in Figures 2(a)–(c) correspond to the depth profile of rapamycin including the background signal from the skin matrix. The depth profiles were vertically adjusted to the same background level for gaining comparability between the treated samples and the control. This was done by subtracting the average of the first 10 data points in the dermis (DE), where no rapamycin is expected to be present in none of the samples and is also justified by the fact that the depth profiles closely overlap at the top region of each scan, where EPON is the only species being present. This adjustment also explains the negative intensity values in these profiles. Note that the reference skin was from the same donor and the stack of micrographs were taken as for the treated samples. However, as there is some variability in skin morphology, the general shape cannot be fully identical to that of the treated skin samples. Region (1) from the 10 min. treated sample, corresponding to a hair follicle, indicates that the shape of the rapamycin profile and that of the background signal are almost identical, but there is a narrow region in the stratum corneum that hints evidence for rapamycin. In contrast, region (2) indicates more clearly in the same depth region of the stratum corneum evidence for rapamycin compared to the background signal. Similar results were also obtained from the 100 min and 1000 min treated samples. Note that the amplitude of the rapamycin signal is always different in magnitude, which is rationalized by different local drug concentrations. This is ascribed to local mechanical damage by microneedle treatment, which is unlike topical treatment of intact skin, where a uniform depth profile is expected, provided the drug can penetrate skin.^[46]^ Interestingly, one could gain the impression that there is some rapamycin present in the viable epidermis reaching below the basal membrane at a constant level, which could be a plausible result of microneedle treatment. However, as the untreated reference sample shows the same depth dependent shape in optical density, rapamycin penetration into the viable parts of skin is discounted, as will be elucidated further below in the context of linear combination modeling and higher spatial resolution STXM‐studies. Therefore, the shape of the background signal reflects a different composition of the viable epidermis and dermis as probed by changes in optical density in the O 1s‐pre‐edge regime.

The amplitudes of the rapamycin signal compared to the background are most narrow for 10 min exposure time (Figure 2(a) regions (1) and (2)), which is ascribed to the lack of bending of this skin layer and a smaller width of the stratum corneum compared to the other regions investigated. A slightly broader spatial drug distribution is found after 100 min, which is partly due to the broader stratum corneum and the slight bending of this skin layer in region (2) of Figure 2(b). The mechanical defect in the entire sample in Figure 2(c) allows us to investigate only a narrow skin region. There, one also observed rapamycin in the stratum corneum with variable amplitude. This result highlights evidence for local variations in drug concentration due to microneedles.

Figure 3 shows with higher spatial resolution and smaller pixel sizes of 250 and 200 nm the distribution of rapamycin for the three samples shown in Figure 2. Figure 3(a)–(c) shows in the top part of Section (1) X‐ray micrographs taken at 532.0 eV, corresponding to exposure times of 10, 100, and 1000 min, respectively. These provide details of the skin morphology, such as the stratified structure of the stratum corneum, consisting of corneocytes and thin lipid layers,^[50]^ as well as round‐shaped structures in the viable epidermis that are due to nuclei of keratinocytes. Figure 3(d) includes results for the untreated reference skin.

The rapamycin distribution is evaluated by two different approaches (i) changes in optical density (bottom part of Section (1)) and (ii) results from linear combination modeling (Sections (2) and (3)). Changes in optical density were derived in the same way as those shown in Figure 2, but slightly different photon energies were used (531.2 eV and 530.2 eV, respectively) in comparison to the overview scans. These results indicate that the choice of photon energies is not critical, especially since there is a background signal due to cross sensitivities from the skin matrix, as shown in Figure 2. The results from linear combination modeling^[12]^ were derived from reference spectra, as shown in Figure S1 (see Supporting Information). These reference spectra were obtained from the maps of the reference skin sample and cover the O 1s‐regime of rapamycin, an average of the skin under study, where the contribution of the embedding medium (EPON) has been subtracted, the embedding medium EPON, as well as an O 1 s→π*‐ and an O 1 s→σ*‐component to account for regions of the skin that contain more unsaturated‐ or more saturated oxygen species. Note that Figures 3 and 4 contain the results for rapamycin, whereas the spatial distributions of the other species are compiled in Figures S5‐S8.

We have derived depth profiles from narrow regions of the spatial rapamycin distribution for avoiding morphology dependent distortions that would occur if the entire region would be used. These regions are indicated by colored rectangles in Figure 3(a) region (1) and are indicated by ‘top’ and ‘bottom’ in all depth profiles of Figure 3. The difference between the Sections (2) and (3) derived from linear combination modeling is described as follows: In Section (2) all reference spectra were normalized to the O 1 s→σ*‐resonance in the 538–540 eV regime to normalize the oxygen content. In Section (3) additionally each pixel of the spectromicroscopy maps was normalized to this spectroscopic feature. The result is that the relative scale corresponding to the rapamycin depth profiles in Sections (3) covers a larger dynamic range while there are minor differences between both normalization procedures with respect to the shape of the depth distribution of the drug. The full importance of this analysis is evident for the high‐spatial resolution scan that are discussed in Figure 4 (see below).

The results indicate independent from the evaluation approach that rapamycin is only found in the stratum corneum. No evidence for the drug is found in the viable epidermis. The spatial distribution of the drug is narrower for linear combination modeling than from changes in optical density. This is ascribed to the cross sensitivities to the skin matrix of the latter approach. The shape of the rapamycin distributions derived from linear combination modeling indicate that the top layers of the stratum corneum do not contain the drug. This points to evidence that the drug is entering the stratum corneum from the mechanically damaged sites, but not topically, as one would expect then to find evidence for rapamycin also in the top layers of the stratum corneum, as shown in previous work.[^11^, ^36^] Note that the rapamycin distributions for the sample that was exposed for 1000 min to the drug formulation shows similar results (Figure 3(c)). However, due to the damage of the entire sample near the stratum corneum this layer is widened and the rapamycin distribution is more structured than for the compact structure of an intact stratum corneum. Finally, reference skin (Figure 3(d)) shows for changes in optical density a broad offset, which is comparable to the results shown in Figure 2.

Linear combination modeling yields weak and noisy contribution of the drug that is rationalized in terms of uncertainties of the fit procedure. We conclude that enhanced spatial resolution provides consistent results compared to the overview scans and changes in optical density, as shown in Figure 2. In addition, more details on the drug penetration process become visible, especially, if depth profiles in narrow regions are evaluated.

Figure 4 covers results from higher resolution images than shown in Figure 3. Here, the focus is the stratum corneum for determining more precisely where rapamycin is located and where its concentration is the highest. Note that in these micrographs not the entire stratum corneum was mapped, so that the depth distribution discussed above is not considered, Rather, the focus is put on evidence for finding rapamycin in the lipid layers separating the corneocytes. Also, for these more detailed scans, the depth profiles of rapamycin were derived for two different narrow regions indicated by dashed rectangles at the top and bottom of each micrograph, as indicated by colored rectangles in Figure 4. This was done for the same reason as explained for Figure 3, i. e., maintaining the spatial resolution of the micrographs in depth profiles. Here, this approach is more relevant, as the narrow lipid layers would be smeared out by integration of wider regions. The same approach was pursued for the untreated reference sample (see Figure 4(d)).

The results indicate for all samples and both data evaluation approaches, except for the untreated reference, that there is a broad continuous background level of rapamycin, which is due to drug‐filled corneocytes. In addition, there are changes in local drug concentration at the lipid layers separating the corneocytes. The lipid layers between corneocytes have a typical thickness of 100–200 nm and can be filled by lipophilic drugs, such as dexamethasone, as long as they do not exceed a molecular weight of 500 Dalton.^[46]^ The X‐ray micrographs indicate by high transmission of the soft X‐rays compared to the corneocytes that the lipid layers are optically thinner than the corneocytes. The local concentration of rapamycin derived from changes in optical density yields significant decreases in the regions of the lipids both for the drug treated and the reference samples, respectively. Due to their natural variability the properties of the lipid layers cannot be easily quantified, as the thickness of the samples is there also slightly lower than for the corneocytes, which follows from additional AFM experiments (see Figure S3). Due to this variability in this part of the skin it is not possible to determine if the drug is contained in these structures using changes in optical density. This indicates inherent limits of this approach for regions that are optically thin and cannot efficiently accommodate the drug due to their small volume. Linear combination modeling yields more distinct information for the spatial distribution of rapamycin if one evaluates the vertical scale of rapamycin abundance. It is for the reference sample smaller than for the drug‐treated samples (see Sections (2) in Figure 4). This situation becomes even more evident, if the pixels of the X‐ray micrographs are also normalized to the O 1 s→σ*‐resonance. This increases the sensitivity for the optically thin lipid layers. The result of this analysis is shown in Sections (3) of Figure 4. Then, there are sharp increases in local rapamycin concentration with enhanced amplitude compared to the reference sample and the background level of drug in the corneocytes at the location of the lipid layers between the corneocytes. These results indicate that even in optically thin regions a highly dilute drug can be probed at the spatial resolution of less than 100 nm, given that all data sets have previously been normalized to compensate for thickness‐ and composition‐dependent variations in optical density. These results indicate, however, that only a small fraction of the drug is bound in these lipids, whereas most of the rapamycin can enter the bulk of the corneocytes, as follows from the low‐resolution X‐ray micrographs.

Previous work on rapamycin penetration facilitated by trypsin treatment and occlusion indicated that the drug is mostly found in the corneocytes.[^11^, ^36^] This was rationalized in terms of enzymatic destruction of the corneocyte envelopes by trypsin and mechanical damage of these structures due to swelling of the corneocytes, respectively. However, these results were unexpected due to the high log P value of 4.3 (cf. ref.^[51]^) of rapamycin that would lead to the expectation that the drug should be found at least in part in the lipids separating the corneocytes, as was found in earlier work on dexamethasone penetration.^[46]^ In the case of microneedle treatment there is also mechanical damage, but different from that resulting from occlusion, as the corneocytes get locally punctured by the microneedles outside the thin investigated samples and allow the rapamycin to enter these structures that are large in their lateral surface compared to their thickness with typical dimensions of 40 μm in length and 0.5 μm in thickness.^[52]^

The density of mechanical damage is sufficiently high for rationalizing the high local concentration of rapamycin within corneocytes as well as the lipid layers (cf. Figures 3 and 4), explaining that primarily the bulk of the corneocytes is filled by rapamycin.

These results imply that the damage caused during the needling process is sufficient to enhance the uptake of rapamycin into the stratum corneum. Such uptake could be beneficial to create depots for prolonged penetration. Yet, the fact that no deeper penetration in viable compartments was observed suggests that the compound in the chosen formulation did not have the capability to translocate from the lipid‐rich environment of the stratum corneum or from within the corneocytes to deeper, more hydrophilic compartments in the viable epidermis.

While the use of longer or finer needles could be considered, their potential mechanical limitations must be taken into account. Alternatively, patch systems incorporating drug‐loaded microneedles that deliver the drug directly during the skin disruption process might offer a promising approach.

## Conclusions

Dermal penetration of the topically applied drug rapamycin that does not penetrate intact human skin due to its high molecular weight is shown to be facilitated by a commercial microneedle device. Rapamycin is selectively probed by tunable soft X‐rays in the O 1s‐regime by scanning transmission X‐ray microscopy. This approach is suitable to gain from spectroscopic signatures selectively the spatial distribution and depth profile of rapamycin by employing two data reduction approaches, i. e., changes in optical density and linear combination modeling, which yield consistent results. Linear combination modeling provides more reliable results for drug distributions since cross sensitivities are weaker than by probing changes in optical density. The results indicate that rapamycin does not reach the viable skin layers and remains in the top horny skin layer, the stratum corneum. There, mostly corneocytes absorb the drug. In addition, in the lipid layers separating the corneocytes, also evidence for the occurrence of lipophilic rapamycin is found. From the therapeutic point of view skin treatment with microneedles after topical drug application opens a reservoir for high molecular weight drugs in the stratum corneum which can be beneficial for prolonged drug treatment.

## Conflict of Interests

The authors declare no conflict of interest.

1

## Data Availability

The data that support the findings of this study are available from the corresponding author upon reasonable request.

## References

[1] A. P. Hitchcock , N. Kosugi , J. Electron Spectrosc. Relat. Phenom. 2024, 266, 147438.

[2] F. M. F. de Groot , E. de Smit , M. M. van Schooneveld , L. R. Aramburo , B. M. Weckhuysen ChemPhysChem. 2010, 11, 951–962. 20306509 10.1002/cphc.200901023

[3] B. O. Leung , J. L. Brash , A. P. Hitchcock , Materials. 2010, 3, 3911–3938. 28883316 10.3390/ma3073911 PMC5445794

[4] B. Graf-Zeiler , R. H. Fink , G. Tzvetkov , ChemPhysChem. 2011, 12, 3503–3509. 21853515 10.1002/cphc.201100370

[5] J. Kim , D. W. Lee , C. Nam , J. K. Y. Chung , B. Koo , N. Kim , J. Lim , J. Electron Spectrosc. Relat. Phenom. 2023, 266, 147337.

[6] K. Yamamoto , R. Flesch , T. Ohigashi , S. Hedtrich , A. Klossek , P. Patoka , G. Ulrich , S. Ahlberg , F. Rancan , A. Vogt , U. Blume-Peytavi , P. Schrade , S. Bachmann , M. Schäfer-Korting , N. Kosugi , E. Rühl , Anal. Chem. 2015, 87, 6173–6179. 25942614 10.1021/acs.analchem.5b00800

[7] J. R. Lawrence , G. D. W. Swerhone , J. J. Dynes , D. R. Korber , A. P. Hitchcock , J. Microsc. 2016, 261, 130–147. 25088794 10.1111/jmi.12156

[8] U. Alexiev , E. Rühl , in Visualization of Nanocarriers and Drugs in Cells and Tissue, Vol. (Eds.: M. Schäfer-Korting , U. S. Schubert ), Springer International Publishing, Cham, 2024, pp.153–189. 10.1007/164_2023_684 37566121

[9] M. A. Marcus , J. Electron Spectrosc. Relat. Phenom. 2023, 264, 147310.

[10] A. P. Hitchcock , J. Electron Spectrosc. Relat. Phenom. 2023, 266, 147360.

[11] G. Germer , T. Ohigashi , H. Yuzawa , N. Kosugi , R. Flesch , F. Rancan , A. Vogt , E. Rühl , J. Electron Spectrosc. Relat. Phenom. 2023, 266, 147343.

[12] G. Germer, Linear Combination Modeling, https://www.wavemetrics.com/node/22023, 2023.

[13] I. N. Koprinarov , A. P. Hitchcock , C. T. McCrory , R. F. Childs , J. Phys. Chem. B. 2002, 106, 5358–5364.

[14] M. Lerotic , R. Mak , S. Wirick , F. Meirer , C. Jacobsen , J. Synchrotron Rad. 2014, 21, 1206–1212. 10.1107/S1600577514013964 25178014

[15] R. Parisi , D. P. M. Symmons , C. E. M. Griffiths , D. M. Ashcroft , J. Invest. Dermatol. 2013, 133, 377–385. 23014338 10.1038/jid.2012.339

[16] D. Y. M. Leung , M. Boguniewicz , M. D. Howell , I. Nomura , O. A. Hamid , J. Clin. Invest. 2004, 113, 651–657. 14991059 10.1172/JCI21060 PMC351324

[17] F. Rancan , M. Giulbudagian , J. Jurisch , U. Blume-Peytavi , M. Calderón , A. Vogt , Eur. J. Pharm. Bipharm. 2017, 116, 4–11. 10.1016/j.ejpb.2016.11.017 27865989

[18] A. Al-Amoudi , J. Dubochet , L. Norlén , J. Invest. Dermatol. 2005, 124, 764–777. 15816835 10.1111/j.0022-202X.2005.23630.x

[19] M. Haftek , M. H. Teillon , D. Schmitt , Microsc. Res. Tech. 1998, 43, 242–249. 9840802 10.1002/(SICI)1097-0029(19981101)43:3<242::AID-JEMT6>3.0.CO;2-G

[20] A. H. Sabri , Y. Kim , M. Marlow , D. J. Scurr , J. Segal , A. K. Banga , L. Kagan , J. B. Lee , Adv. Drug Deliv. Rev. 2020, 153, 195–215. 31634516 10.1016/j.addr.2019.10.004

[21] K. van der Maaden , W. Jiskoot , J. Bouwstra , J. Control. Release. 2012, 161, 645–655. 22342643 10.1016/j.jconrel.2012.01.042

[22] E. Larrañeta , M. T. C. McCrudden , A. J. Courtenay , R. F. Donnelly , Pharm. Res. 2016, 33, 1055–1073. 26908048 10.1007/s11095-016-1885-5 PMC4820498

[23] E. Larrañeta , R. E. M. Lutton , A. D. Woolfson , R. F. Donnelly , Mater. Sci. Engin. Rep. 2016, 104, 1–32.

[24] M. R. Prausnitz , Annu. Rev. Chem. Biomol. Eng. 2017, 8, 177–200. 28375775 10.1146/annurev-chembioeng-060816-101514

[25] T. Waghule , G. Singhvi , S. K. Dubey , M. M. Pandey , G. Gupta , M. Singh , K. Dua , Biomed. Parmacotherap. 2019, 109, 1249–1258. 10.1016/j.biopha.2018.10.078 30551375

[26] K. A. S. Al-Japairai , S. Mahmood , S. H. Almurisi , J. R. Venugopal , A. R. Hilles , M. Azmana , S. Raman , Int. J. Pharm. 2020, 587, 119673. 32739388 10.1016/j.ijpharm.2020.119673 PMC7392082

[27] T. T. Peng , Y. Y. Chen , W. S. Hu , Y. Huang , M. M. Zhang , C. Lu , X. Pan , C. B. Wu , Engineering. 2023, 30, 170–189.

[28] R. Habib , A. K. Azad , M. Akhlaq , F. A. Al-Joufi , G. Shahnaz , H. R. H. Mohamed , M. Naeem , A. S. A. Almalki , J. Asghar , A. Jalil , M. M. Abdel-Daim , Polymers. 2022, 14, 415. 35160403 10.3390/polym14030415 PMC8839939

[29] J. H. Lee , M. A. Neustrup , B. Slütter , C. O'Mahony , J. A. Bouwstra , K. van der Maaden , Pharm. Res. 2024, 41, 305–319. 38332390 10.1007/s11095-024-03665-7 PMC10879229

[30] Z. Sartawi , C. Blackshields , W. Faisal , J. Control. Release. 2022, 348, 186–205. 35662577 10.1016/j.jconrel.2022.05.045

[31] Z. F. Rad , P. D. Prewett , G. J. Davies , Beilstein J. Nanotechnol. 2021, 12, 1034–1046. 34621614 10.3762/bjnano.12.77 PMC8450954

[32] S. Bhatnagar , K. Dave , V. V. K. Venuganti , J. Control. Release. 2017, 260, 164–182. 28549948 10.1016/j.jconrel.2017.05.029

[33] R. J. Motzer , B. Escudier , S. Oudard , T. E. Hutson , C. Porta , S. Bracarda , V. Grünwald , J. A. Thompson , R. A. Figlin , N. Hollaender , G. Urbanowitz , W. J. Berg , A. Kay , D. Lebwohl , A. Ravaud , Lancet. 2008, 372, 449–456.18653228

[34] A. Peramo , C. L. Marcelo , Arch. Dermatol. Res. 2013, 305, 163–171. 22960739 10.1007/s00403-012-1288-3

[35] A. W. Swarbrick , A. J. Frederiks , R. S. Foster , Austral. J. Dermatol. 2021, 62, 461–469. 10.1111/ajd.13671 34328215

[36] G. Germer , T. Ohigashi , H. Yuzawa , N. Kosugi , R. Flesch , F. Rancan , A. Vogt , E. Rühl , ACS Omega. 2021, 6, 12213–12222. 34056375 10.1021/acsomega.1c01058 PMC8154144

[37] J. D. Bos , M. M. H. M. Meinardi , Exp. Dermatol. 2000, 9, 165–169. 10839713 10.1034/j.1600-0625.2000.009003165.x

[38] N. Kirschner , J. M. Brandner , Ann. N. Y. Acad. Sci. 2012, 1257, 158–166. 22671602 10.1111/j.1749-6632.2012.06554.x

[39] A. Kubo , K. Nagao , M. Yokouchi , H. Sasaki , M. Amagai , J. Exp. Med. 2009, 206, 2937–2946. 19995951 10.1084/jem.20091527 PMC2806471

[40] R. Schulz , K. Yamamoto , A. Klossek , R. Flesch , S. Hönzke , F. Rancan , A. Vogt , U. Blume-Peytavi , S. Hedtrich , M. Schäfer-Korting , E. Rühl , R. R. Netz , Proc. Nat. Acad. Sci. U. S. A. 2017, 114, 3631–3636. 10.1073/pnas.1620636114 PMC5389326 28320932

[41] R. Schulz , K. Yamamoto , A. Klossek , F. Rancan , A. Vogt , C. Schütte , E. Rühl , R. R. Netz , Biophys. J. 2019, 117, 998–1008. 31400921 10.1016/j.bpj.2019.07.027 PMC6732523

[42] F. J. Verbaan , S. M. Bal , D. J. van den Berg , W. H. H. Groenink , H. Verpoorten , R. Lüttge , J. A. Bouwstra , J. Control. Release. 2007, 117, 238–245. 17196697 10.1016/j.jconrel.2006.11.009

[43] B. L. Henke , E. M. Gullikson , J. C. Davis , At. Data Nucl. Data Tab. 1993, 54, 181–342.

[44] Auto-Stamp Motorized Meso Machine, https://www.bellezastars.com/product/auto-stamp-motorized-meso-machine/, **2024**.

[45] K. Yamamoto , A. Klossek , R. Flesch , T. Ohigashi , E. Fleige , F. Rancan , J. Frombach , A. Vogt , U. Blume-Peytavi , P. Schrade , S. Bachmann , R. Haag , S. Hedtrich , M. Schäfer-Korting , N. Kosugi , E. Rühl , J. Control. Release. 2016, 242, 64–70. 27568290 10.1016/j.jconrel.2016.08.028

[46] K. Yamamoto , A. Klossek , A. Flesch , F. Rancan , M. Weigand , I. Bykova , M. Bechtel , S. Ahlberg , A. Vogt , U. Blume-Peytavi , P. Schrade , S. Bachmann , S. Hedtrich , M. Schäfer-Korting , E. Rühl , Eur. J. Pharm. Biopharm. 2017, 118, 30–37. 27998691 10.1016/j.ejpb.2016.12.005

[47] K. Yamamoto , A. Klossek , K. Fuchs , B. Watts , J. Raabe , R. Flesch , F. Rancan , H. Pischon , M. Radbruch , A. D. Gruber , L. Mundhenk , A. Vogt , U. Blume-Peytavi , P. Schrade , S. Bachmann , R. Gurny , E. Rühl , Eur. J. Pharm. Biopharm. 2019, 139, 68–75. 30849430 10.1016/j.ejpb.2019.03.006

[48] B. Wanjiku , K. Yamamoto , A. Klossek , F. Schumacher , H. Pischon , L. Mundhenk , F. Rancan , M. M. Judd , M. Ahmed , C. Zoschke , B. Kleuser , E. Rühl , M. Schäfer-Korting , Anal. Chem. 2019, 91, 7208–7214. 31090401 10.1021/acs.analchem.9b00519

[49] G. Germer, Dissertation, Freie Universität Berlin, **2022**.

[50] P. M. Elias , J. Invest. Dermatol. 2012, 132, 2131–2133. 22895445 10.1038/jid.2012.246 PMC3587970

[51] Drug, Bank, Rapamycin, Drug Bank, https://www.drugbank.ca/indications/DBCOND0086651, **2023**.

[52] P. Garidel , Phys. Chem. Chem. Phys. 2002, 4, 5671–5677.

